# Astaxanthin Protects Steroidogenesis from Hydrogen Peroxide-Induced Oxidative Stress in Mouse Leydig Cells

**DOI:** 10.3390/md13031375

**Published:** 2015-03-16

**Authors:** Jyun-Yuan Wang, Yue-Jia Lee, Mei-Chia Chou, Renin Chang, Chih-Hsien Chiu, Yao-Jen Liang, Leang-Shin Wu

**Affiliations:** 1 Department of Animal Science and Technology, College of Bio-Resources and Agriculture, National Taiwan University, Taipei 106, Taiwan; E-Mails: jyunyuanwang@gmail.com (J.-Y.W.); moon7bee@gmail.com (Y.-J.L.); chiuchihhsien@ntu.edu.tw (C.-H.C.); 2 Department and Institute of Life Science, Fu-Jen Catholic University, New Taipei City 242, Taiwan; E-Mail: 071558@mail.fju.edu.tw; 3 Department of Physical Medicine and Rehabilitation, Kaohsiung Veterans General Hospital, Pingtung Branch, Pingtung 912, Taiwan; E-Mail: meeichia@yahoo.com.tw; 4 Department of Emergency Medicine, Kaohsiung Veterans General Hospital, Kaohsiung 813, Taiwan; E-Mail: rnchang@vghks.gov.tw

**Keywords:** astaxanthin, steroidogenesis, testosterone, oxidative stress, hydrogen peroxide

## Abstract

Androgens, especially testosterone produced in Leydig cells, play an essential role in development of the male reproductive phenotype and fertility. However, testicular oxidative stress may cause a decline in testosterone production. Many antioxidants have been used as reactive oxygen species (ROS) scavengers to eliminate oxidative stress to protect steroidogenesis. Astaxanthin (AST), a natural extract from algae and plants ubiquitous in the marine environment, has been shown to have antioxidant activity in many previous studies. In this study, we treated primary mouse Leydig cells or MA-10 cells with hydrogen peroxide (H_2_O_2_) to cause oxidative stress. Testosterone and progesterone production was suppressed and the expression of the mature (30 kDa) form of StAR protein was down-regulated in MA-10 cells by H_2_O_2_ and cAMP co-treatment. However, progesterone production and expression of mature StAR protein were restored in MA-10 cells by a one-hour pretreatment with AST. AST also reduced ROS levels in cells so that they were lower than the levels in untreated controls. These results provide additional evidence of the potential health benefits of AST as a potential food additive to ease oxidative stress.

## 1. Introduction

Androgen plays an essential role in the development of the male reproductive phenotype and fertility. More than 95% of circulating androgens are derived from the testis while the remainder is primarily produced by the adrenals. The major androgen, testosterone, stimulates the embryo to differentiate into a male before birth, and also regulates fertility, promoting the development of male secondary sexual characteristics after birth [1]. Generally, production of testosterone by testicular Leydig cells is controlled by luteinizing hormone (LH), a tropic hormone released from the pituitary. Through its G protein coupled receptor, LH activates adenylyl cyclase by stimulating G protein and inducing production of the intracellular second messenger cAMP, followed by activation of the PKA pathway and promotion of the expression of key steroidogenic genes. These genes include steroidogenic acute regulatory protein (*StAR*), which transports cholesterol into mitochondria, and *Cyp11a1*, which cleaves the side chain of cholesterol and catalyzes its conversion into pregnenolone (P_5_). However, the concentration of testosterone in an individual is easily affected by many physiological and biochemical factors. Disturbance of testosterone synthesis or its functions may cause incomplete masculinization before puberty and many reproductive disorders, including poor sperm production, poor sperm quality, azoospermia, cryptorchidism, hypospadias, and even testicular cancer [2].

Oxidative stress is one of the major factors that reduce production of testosterone in Leydig cells. Oxidative stress results from an imbalance between production of reactive oxygen species (ROS) and the scavenging ability of cellular antioxidant defense systems. ROS, including oxygen singlet, superoxide anion (O_2_·^−^), hydrogen peroxide (H_2_O_2_), and hydroxyl radical (OH·^−^), are oxidizing agents. Excessive ROS production may cause DNA damage, protein damage, and lipid peroxidation, which principally alters the membrane structure and function [3]. During a testicular infection, bacterial lipopolysaccharide (LPS)-induced oxidative stress also alters steroidogenesis and spermatogenesis [4]. Multiple studies indicate that ROS alters testosterone production by reducing StAR protein expression and the activity of HSD3B1 and CYP11A1. Because the mitochondria are the critical site for steroid hormone biosynthesis, dissipation of mitochondrial membrane potential caused by ROS also causes down-regulation of testicular testosterone [5,6,7].

Many antioxidants have been described as ROS scavengers that protect steroidogenesis and/or sperm quality in the testis, avoiding infertility resulting from low testosterone production due to excessive ROS. For example, both ascorbic acid and α-tocopherol (Vitamin C and E) can reverse the reduction of testicular testosterone level caused by oxidative damage by rescuing the deficiencies of steroidogenic enzyme activity [8,9]; however, these antioxidants only partially recover the normal level of testosterone. In the present study, by using Leydig cells treated with H_2_O_2_ as a model of oxidative stress, we aim to test the ability of a stronger antioxidant, astaxanthin (AST), to protect steroidogenesis in Leydig cells from damage by oxidative stress.

**Figure 1:**
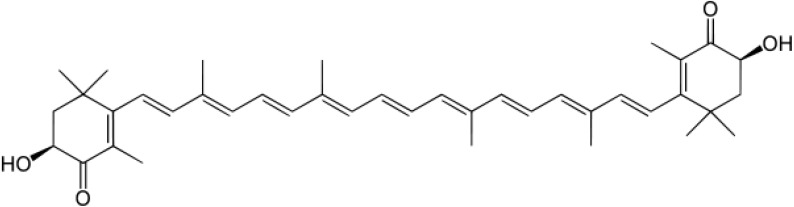
The molecular structure of astaxanthin (AST) is shown.

AST, a red-orange xanthophyll carotenoid, is a natural extract from algae and plants. AST is ubiquitous in the marine environment and can also be found in animals like crabs, salmon, and shrimp. In addition to being found in a variety of living organisms, research has indicated that AST has many diverse clinical benefits and is high in antioxidant activity [10,11,12]. As an antioxidant AST quenches free radicals or other oxidants and protects the lipid bilayer from peroxidation with its polar ionic rings and non-polar conjugated carbon-carbon bonds [13,14]. Due to its unique chemical structure (Figure 1), the antioxidant property of AST is about 10-fold greater than those of other carotenoids, including lutein, canthaxanthin, and β carotene [15]. AST has been approved as a nutraceutical by The United States Food and Drug Administration since 1999. [16]. Now, this antioxidant is available as a commercial nutritional supplement in USA, Sweden, Japan, South Korea, and Taiwan. Due to its safety and outstanding capacity to eliminate free radicals, AST has already become a popular antioxidant added to food and nutritional supplements.

Because there are many published studies focusing on the effect of AST on maintenance of cellular function under oxidative stress, in the present report we aim to determine whether AST can rescue testosterone production under the oxidative stress caused by H_2_O_2_ and to describe its mechanism of action.

## 2. Results

### 2.1. Astaxanthin (AST) Rescues Progesterone and Testosterone Secretion from Testicular Leydig Cells Reduced by Oxidative Stress

To clarify the potential effects of AST, after pre-treatment with 10 μg/mL AST for 1 h, MA-10 cells were treated with 100 μM 8-Br-cAMP, 1 μg/mL 22R-OHC, or 1 μg/mL pregnenolone to stimulate progesterone production and co-treated with 100 μM H_2_O_2_ for an additional 3 h to establish an oxidative stress while the cells were still being exposed to AST they were co-treated with 100 μM H_2_O_2_ for an additional 3 h to establish an oxidative stress.

**Figure 2:**
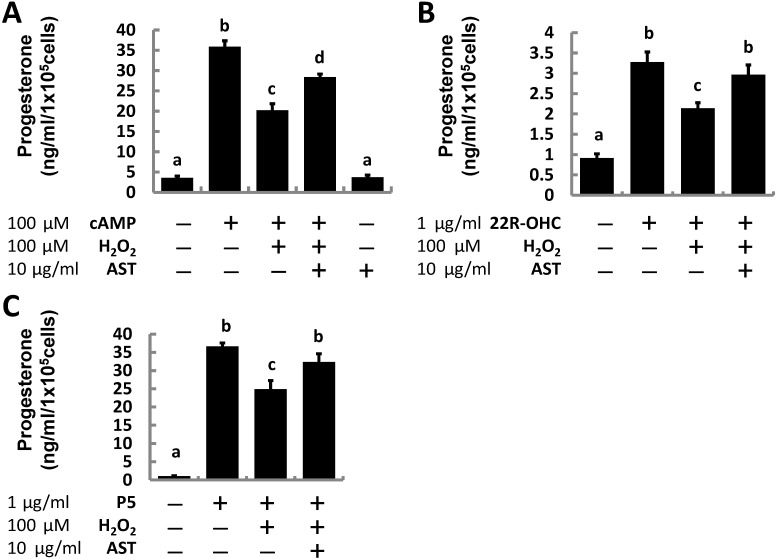
Astaxanthin (AST) restored progesterone production in MA-10 cells induced by cAMP and steroid precursors (22R-OHC, pregnenolone) inhibited by hydrogen peroxide. Cells exposed to AST for 1 h and untreated controls were challenged with 100 μM hydrogen peroxide and (**A**) 100 μM Camp; (**B**) 1 μg/mL 22R-OHC; or (**C**) 1 μg/mL pregnenolone (P_5_) for additional 3 h (*n* = 3). Data were shown as mean with SEM. Bars labeled with different letters (a–d) are significantly different (*p* < 0.05) from each other.

Progesterone production in the MA-10 cells was increased by 10-fold after treatment with cAMP (100 μM), but only six-fold when co-treated with cAMP and H_2_O_2_ (19.47 ng/mL), suggesting that H_2_O_2_ suppressed progesterone production. Although AST alone did not promote steroidogenesis, the antioxidant rescued progesterone production supported by cAMP to 25.76 ng/mL in the presence of H_2_O_2_ (Figure 2A). We also found that even progesterone production supported by exogenous steroidogenic substrates including 22R-OHC and pregnenolone was suppressed under the oxidative stress induced by H_2_O_2_ treatment. Pretreating cells with AST for an additional 1 h partially protected the substrate-supported steroidogenesis in MA-10 cells under oxidative stress (Figure 2B,C).

**Figure 3:**
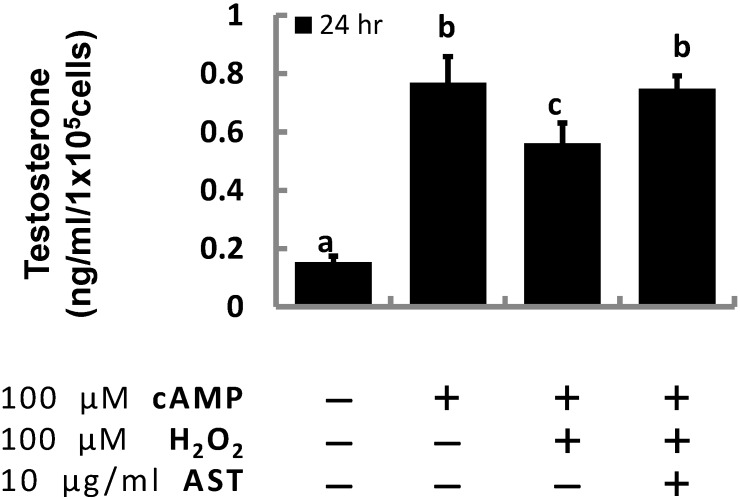
Astaxanthin (AST) rescued the increased testosterone production in primary Leydig cells induced by cAMP impaired by hydrogen peroxide. Cells exposed to AST for 1 h or untreated controls were challenged with 100 μM cAMP, 100 μM hydrogen peroxide, or both for an additional 23 h (*n* = 3). Data were shown as mean with SEM. Bars labeled with different letters (a–c) are significantly different (*p* < 0.05) from each other.

To corroborate our previous results, we examined the effect of AST on testosterone production in primary Leydig cells isolated from adult mouse testes. Similar to the results in the MA-10 cell experiment, cAMP-induced testosterone production (0.77 ng/mL) was roughly five-fold greater than that of the vehicle group (0.15 ng/mL); whereas, the induced production was significantly decreased after cells were co-treated with H_2_O_2_ (0.56 ng/mL). However, pre-treatment with AST kept testosterone production (0.75 ng/mL) at the same level as the cAMP-treated group (Figure 3).

These results suggest that AST supplementation could rescue lost progesterone production resulting from the addition of H_2_O_2_ in both a Leydig cell line and primary mouse Leydig cells.

### 2.2. AST Reduced ROS Levels in MA-10 Leydig Cells

Previous results indicated that steroidogenesis in MA-10 cells is protected by AST under oxidative stress induced by H_2_O_2_. To determine if the effect of AST on steroidogenesis is through scavenging elevated ROS within the cells 2',7'-dichlorodihydrofluorescein diacetate (DCFH-DA), a probe that converts into the highly fluorescent 2',7'-dichlorofluorescein (DCF) after being oxidized by ROS, was used to analyze ROS levels in MA-10 Leydig cells.

**Figure 4:**
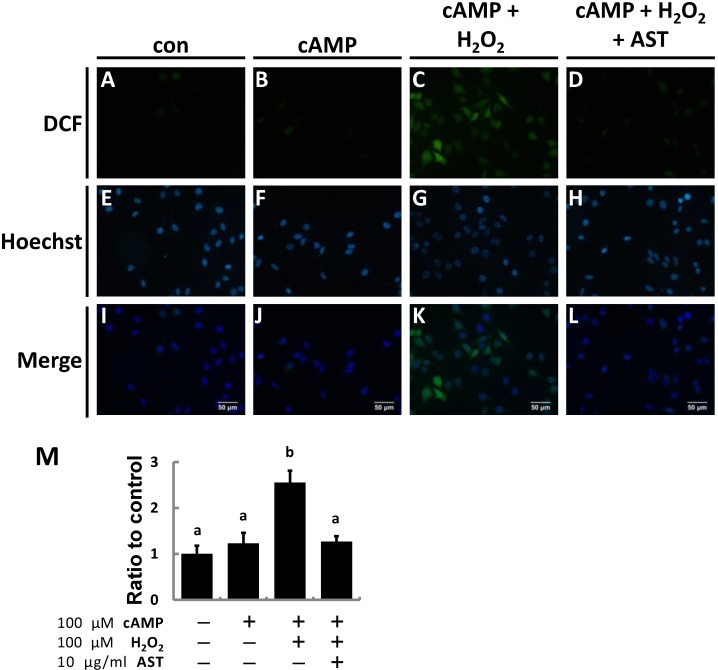
Astaxanthin (AST) protects MA-10 Leydig cells from peroxide-induced ROS. After treatments, cells were incubated in the medium containing 10 μM DCFH-Da for 30 min following a one-minute incubation with 10 μg/mL Hoechst. (**A**–**L**) Treated cells on the slides were visualized using fluorescence microscopy. Images from top to bottom display produced ROS (green), nuclei (blue), and a merged image of the previous two; (**M**) DCF/Hoechst fluorescence was analyzed by microplate fluorometry using excitation and emission wavelengths of 485 (355) nm and 530 (465) nm, respectively. Data were shown as mean with SEM. Bars labeled with different letters (a,b) are significantly different (*p* < 0.05, *n* = 3) from each other.

After treatment with 8-Br-cAMP, 22R-OHC, or pregnenolone MA-10 cells were stained with 10 μM DCFH-DA for 30 min followed by 10 μg/mL Hoechst for 1 min. Exposure to cAMP did not increase the fluorescence intensity compared to controls; however, co-treatment of cAMP and H_2_O_2_ significantly elevated the ROS level in MA-10 cells. Administration of AST 1 h before cAMP and H_2_O_2_ co-treatment could vigorously eliminate ROS in cells, described as reduced green fluorescence intensity as shown in the right column of Figure 4A. The fluorescence microscopy findings were quantified (Figure 4B) and the relative ROS production in the cells is represented as the ratio of fluorescence of DCF and Hoechst staining. Because ROS production only increased by 20% in cAMP-treated cells, ROS level rose to over two-fold production of the control group due to addition of H_2_O_2_. Nevertheless, ROS production in the cells pre-treated with AST was the same as that in the cells treated with only cAMP. Briefly, both the fluorescence images and statistical data confirmed that AST could scavenge ROS in the cells under oxidative stress. We postulate that attenuating the production of ROS or eliminating ROS may be possible mechanisms involved in the protection of steroidogenesis by AST.

### 2.3. AST Protects the Expression Level of Steroidogenic Proteins (StAR, P450scc, and 3β-HSD) in MA-10 Leydig Cells during Oxidative Stress

**Figure 5:**
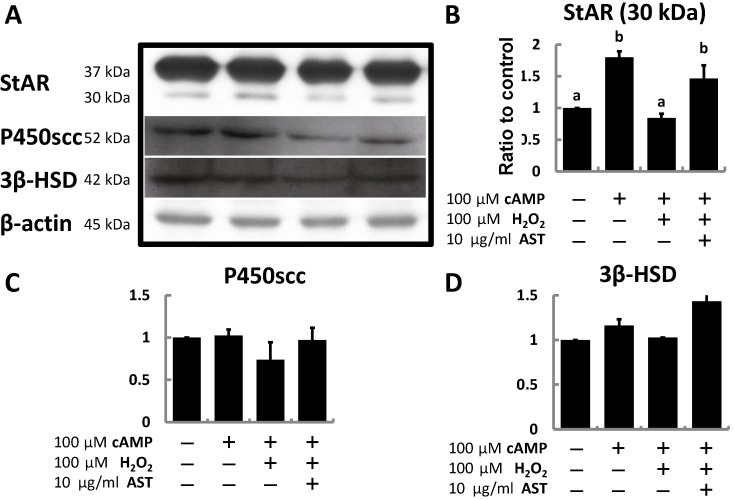
Astaxanthin restores the expression of steroidogenic proteins (StAR, P450scc, and 3β-HSD) in MA-10 cells inhibited by peroxide. (**A**) Representative western blot analysis of MA-10 cells for StAR, P450scc, 3β-HSD proteins, and β-actin loading controls (30 μg of total protein/lane). The relative values (ratio to β-actin) of (**B**) 30 kDa StAR; (**C**) P450scc; and (**D**) 3β-HSD were quantified by densitometric analysis (*n* = 3). Data were shown as mean with SEM. Different symbols (a,b) indicate a significant difference (*p* < 0.05) from each other.

Finally, MA-10 cells were treated with cAMP and H_2_O_2_ for 3 h or treated with AST for 4 h with co-treatment with cAMP and H_2_O_2_ during the last 3 h to evaluate the effect of H_2_O_2_ and AST on steroidogenic protein expression. After treatment, only the expression of the 30 kDa isoform of StAR protein was significantly induced by cAMP (1.8-fold higher than controls), and the increased expression level was totally eliminated by H_2_O_2_. However, with pre-treatment by AST, the expression level of StAR didn’t obviously decrease (Figure 5B). In contrast, neither P450scc nor 3β-HSD was significantly regulated by cAMP, H_2_O_2_, or AST (Figure 5C,D).

## 3. Discussion

Over the past several decades, numerous studies have described the biological functions and clinical benefits of AST [17], especially in protection against oxidation by ROS [10,18,19]. Because of its antioxidant ability and other beneficial functions, the United States Food and Drug Administration approved AST as a nutraceutical in 1999 [16]. Recently, AST has been described to improve sperm quality and function during redox system imbalance [20]. Moreover, Comhaire and his colleagues found that AST decreases ROS and the secretion of inhibin B by Sertoli cells, also enhancing the pregnancy rate in a double-blind randomized trial [21]. Thus, further exploration whether AST also protects the endocrine system required for reproductive behavior and production of normal fertile testicular spermatozoa may be worthwhile.

Steroid hormones are synthesized through steroidogenesis in the cells of the adrenal cortex and the gonads (testis and ovary). Steroidogenesis in the testis and ovary are mainly regulated by LH; whereas, this process is controlled by ACTH, a tropic hormone released from anterior pituitary, in the adrenal [22]. LH and ACTH bind to their membrane-bound G protein-coupled receptors and signal by activating the cAMP/PKA pathway, the major signaling pathway regulating steroid biosynthesis [23].

Hydrogen peroxide has a negative effect on steroidogenesis. *In vivo* generation of hydrogen peroxide occurs during luteolysis in the superovulated rat in response to prostaglandin F2α secretion from the rat corpus luteum [24]. Under hydrogen peroxide treatment estradiol and progesterone secretion are inhibited in human luteal cells [25]. The acute blockade of steroidogenesis may be due to uncoupling of LH receptor and adenylyl cyclase [26] and the effect of suppressed expression of CYP11A1 on cholesterol utilization [27]. Furthermore, LDL uptake by the porcine luteal cell is inhibited by hydrogen peroxide [28]. Oxidative stress induced by hydrogen peroxide, 4-hydroxy-2-nonenal (HNE), or thiobaribituric acid-reactive substances (TBARS) also suppresses steroid hormone production in adrenal cells by activating the p38 MAPK pathway [29,30]. Hydrogen peroxide suppresses steroidogenesis in MA-10 cells [6,31], and hydrogen peroxide decreases the mature form of StAR protein but not its mRNA expression [5,7]. Oxidative damage caused by hydrogen peroxide not only reduces the expression level of StAR protein, but also disrupts mitochondrial membrane potential (ΔΨm) [7]. In the mouse Leydig cell line K28 JNK is responsible for the suppression of CYP17A1 expression by decreasing the transcriptional activity of NR4A1 [32].

In the present study, progesterone production in MA-10 cells is significantly increased by 10-fold after cells are treated with 8-Br-cAMP. However, this elevated production of progesterone is reduced by half when the cells are co-treated with cAMP and hydrogen peroxide, similar to previous studies. Co-treatment with AST diminishes the effect of hydrogen peroxide and restores steroidogenesis. The same effect can be seen when MA-10 cells are treated with 22-hydroxycholesterol or pregnenolone, in which hydrogen peroxide also suppresses progesterone production supported by these two steroid substrates.

Not only can hydrogen peroxide suppress progesterone production in the immortalized MA-10 Leydig cell-like line, but also in primary mouse Leydig cells. Treatment of primary rat Leydig cells with polychlornated biphenyls (PCBs) promotes ROS formation and reduces the activity of enzymes necessary for testosterone production [33]. A similar effect can be seen in this study since 22R-hydroxycholesterol is catalyzed into progesterone by the activity of CYP11A1 and HSD3B1 while pregnenolone is converted into progesterone by HSD3B1. Inhibition of progesterone production demonstrates that the enzymatic activities of CYP11A1 and HSD3B1 are inhibited under oxidative stress.

To prove that hydrogen peroxide enhances ROS production in MA-10 cells, DCFH-Da was used to indicate intracellular ROS level. Based on our results, cAMP does not significantly alter the cellular ROS level. As expected, hydrogen peroxide increases the ROS level while co-treatment with AST attenuates the ROS level in MA-10 cells. These results clearly demonstrate that AST can be a scavenger of ROS, can decrease oxidative stress on MA-10 cells, and can also restore steroidogenesis in these cells.

Finally, since hydrogen peroxide attenuates the post-cAMP pathway, mRNA or protein expression of steroidogenic genes may be altered under by this treatment. The expression of the mature form of StAR (30 kDa) was decreased by hydrogen peroxide treatment, but was restored after co-treatment with AST.

## 4. Experimental Section

### 4.1. Reagents and Chemicals

Cell culture medium DMEM/F12 and medium 199, trypsin-EDTA (0.25%), penicillin G, streptomycin sulfate, fetal bovine serum (FBS), Hank’s balanced salt solution (HBSS), and trypan blue stain were purchased from Invitrogen Corporation (Grand Island, NY, USA). Collagenase type 1 was purchased from Worthington Biochemical Corporation (Lakewood, NJ, USA). Bovine serum albumin (BSA) and other general chemicals were purchased from Sigma-Aldrich (St. Louis, MO, USA), and 8-bromocyclic AMP was purchased from Tocris Bioscience (Ellisville, MO, USA). Pregnenolone was purchased from Steraloids, Inc. (Newport, RI, USA) and 22-hydroxycholesterol (22ROHC) was purchased from Sigma-Aldrich.

### 4.2. Primary Mouse Leydig Cell Culture

Male C57BL/6 mice (5-week-old) were purchased from National Taiwan University, maintained under 12-h light (0900–2100)/12-h dark (2100–0900) conditions, and allowed free access to chow and water. All mice were retained until 10-weeks-old before isolating primary Leydig cells. All experimental protocols were approved by the Animal Care and Use Committee in the College of Medicine, National Taiwan University. All procedures conformed to the National Institutes of Health Guide for the care and use of laboratory animals. After mice were sacrificed by decapitation, the testes were removed and decapsulated in medium 199 supplemented with 10% serum. Then, the seminiferous tubules were separated, washed once with isolation buffer (10 mL HBSS containing 0.1% BSA and 200 U/mL collagenase type 1), and incubated at room temperature in isolation buffer for an additional 5 min. The seminiferous tubules and cells were filtered through a 250 micron mesh, and then the cells were collected by centrifugation at 300 × *g* for 5 min and resuspended in 10 mL of medium 199 without supplemental serum. Live cells were counted with trypan blue stain, and then seeded (10^6^ cells per mL) in culture tubes (Corning Inc, Oneonta, NY, USA). Cells were immediately treated with different doses of cAMP, H_2_O_2_ and AST and incubated at 37 °C with 5% CO_2_. After 4 h, the culture medium was collected and stored at −20 °C until the enzyme immunoassay (EIA) was performed.

### 4.3. Culture of MA-10 Cells

MA-10 mouse Leydig tumor cells were seeded into T-75 cell culture flasks (Thermo Scintific, West Palm Beach, FL, USA) with DMEM/F-12 medium, supplemented with 10% FBS, 2.2 mg/mL sodium bicarbonate, 100 U/mL penicillin, and 0.1 mg/mL streptomycin and incubated at 37 °C with 5% CO_2_. Before experiments, nonadherent cells were removed by aspiration, and healthy cells were collected by trypsinization and centrifugation. The cell pellets were gently resuspended in DMEM/F-12 medium without FBS, and cells were seeded into 48-well plates (5 × 10^4^ cells/well), after incubation overnight, medium was changed and all cell treated with different chemicals, and incubated at 37 °C with 5% CO_2_. After treatment, the culture medium was collected and stored at −20 °C until EIA was performed.

### 4.4. Enzyme Immunoassay for Progesterone and Testosterone

The progesterone assay was modified from a direct EIA using G7, an IgM monoclonal antibody with specific affinity of 1.1 × 10^10^ M, as described previously [34,35,36]. G7 exhibits cross-reactivity of <0.01% with BSA and other steroids including pregnenolone, testosterone, estradiol, and estrone. Aliquots (50 μL) of diluted medium and horseradish peroxide-linked progesterone conjugate (150 μL) were added to microtiter plates coated with 200 μL G7 (representing a 1:40,000 dilution). After a 15-min incubation at room temperature with gentle shaking and two washes with Tween-20 in 0.01 M phosphate buffer (pH 7.0), the color was developed with 3.7 mM O-phenylenediamine (200 μL) in 0.03% H_2_O_2_ for an additional 15 min. The reaction was stopped by adding 8N H_2_SO_4_ (50 μL). The optical density (OD) of each sample was determined with μQuant EIA reader (BioTek, Winooski, VT, USA) set wavelength of absorption between 490 and 630 nm. Progesterone concentration was determined with a standard curve. Coefficients of variation were 7% (within assays) and 12% (between assays). The sensitivity of the assay was 0.3 pg/mL [35]. All standards and samples were assayed in duplicate.

### 4.5. Staining of Intracellular ROS of MA-10 Cells

Method of ROS staining was modified from previous study [37]. Briefly, MA-10 cells were seeded on round cover slides, which were put in a 24-well cultured plate. After treated with AST, cAMP and H_2_O_2_, medium were removed and cells were washed once with DMEM/F12 medium without serum then incubated in medium containing 10 μM 2',7'-dichlorodihydrofluorescein diacetate (DCFH-DA) for 30 min following 1 min incubation with 10 μg/mL Hoechst 33342. After treatment, cover slides were removed from 24-well dish and washed with PBS and cover on a glass slide for fluorescent microscope observation (Olympus IX-70 with fluorescent facility). Images were captured with camera (Olympus E-330, Olympus, Shinjuku, Japan). For quantitative assay, MA-10 cells were seeded in black 96-well plate. After treated with AST, cAMP, H_2_O_2_ and stained with DCFH-DA and Hoechst 33342, plate was read with microplate reader Synergy H1 (BioTek) with excitation of 485 nm, emission of 530 nm for DCF and excitation of 355 nm, emission of 465 nm for Hoechst 33342.

### 4.6. Western Blot Analysis

After treatment, the cells were rinsed twice with cold phosphate-buffered saline (PBS) and harvested. Then the cells were resuspended in cold lysis buffer (2% SDS, 50mM Tris (pH 6.8), 5mM EDTA, 1% 2-mercaptoethanol, 5% glycerol), and whole-cell extracts were prepared, as described previously [38]. Samples containing 40 μg protein were separated by 12% SDS-polyacrylamide gel electrophoresis, as described previously [38]. The separated proteins were transferred to a polyvinylidene fluoride membrane. The membrane was blocked by incubating in PBS containing 0.01% Tween-20 (PBST) and 2.5% BSA for 8 h at room temperature, followed by incubation with rabbit polyclonal primary antibodies specific for StAR (1:10,000 dilution) [36,39], CYP11A1 (1:5000 dilution), HSD3B1 (1:10,000 dilution) [36,40], and ACTB (1:5000 dilution) (MAB1501, Millipore, Temecula, CA, USA) in PBST overnight at 4 °C. After four washes with PBST, the membrane was incubated for 2 h with peroxidase conjugated goat anti-rabbit IgG (1:7000 dilution; Jackson ImmunoResearch, West Grove, PA, USA). The membranes were washed with PBST, and bound antibodies were visualized by the ECL system (Millipore) using Kodak X-OMAT film (Eastman Kodak Co., Rochester, NY, USA).

### 4.7. Data Analysis

Western blot experiments were performed three times, and a representative result is shown. Densities of blots were quantified with VisionWorksLS Image Acquisition and Analysis Software (UVP, Upland, CA, USA). Results are presented as multiple of control. Results are expressed as mean with standard error of the mean (SEM) of triplicate samples from three individual experiments. Western blot data were analyzed with Student’s *t*-test using Microsoft Excel 2007. Results of other assays were analyzed by one-way ANOVA followed by Duncan’s multiple comparison using SigmaStat 12 (Aspire Software International, Ashburn, VA, USA). *p* < 0.05 was considered significant.

## 5. Conclusions

To our knowledge, this is the first study to demonstrate that AST can protect steroidogenesis in Leydig cells from oxidative stress. Hydrogen peroxide can induce oxidative stress and attenuate the post-PKA pathway resulting in suppressed expression of the mature form of StAR protein. However, by reducing ROS formation caused by hydrogen peroxide, AST prevents the down-regulation of the mature form of the StAR protein, and restores steroidogenesis in the Leydig cell.
